# Microbiological Quality of Ready-to-Eat Vegetables Collected in Mexico City: Occurrence of Aerobic-Mesophilic Bacteria, Fecal Coliforms, and Potentially Pathogenic Nontuberculous Mycobacteria

**DOI:** 10.1155/2015/789508

**Published:** 2015-03-30

**Authors:** Jorge Francisco Cerna-Cortes, Nancy Leon-Montes, Ana Laura Cortes-Cueto, Laura P. Salas-Rangel, Addy Cecilia Helguera-Repetto, Daniel Lopez-Hernandez, Sandra Rivera-Gutierrez, Elizabeth Fernandez-Rendon, Jorge Alberto Gonzalez-y-Merchand

**Affiliations:** ^1^Departamento de Microbiologia, Escuela Nacional de Ciencias Biologicas-Instituto Politecnico Nacional, Prolongacion Carpio y Plan de Ayala S/N, Colonia Casco de Santo Tomas, Delegacion Miguel Hidalgo, 11340 Mexico, DF, Mexico; ^2^Departamento de Inmunobioquimica, Torre de Investigación, Instituto Nacional de Perinatología Isidro Espinosa de los Reyes (INPer), Montes Urales 800, Colonia Lomas de Virreyes, 11000 Mexico, DF, Mexico; ^3^Departamento de Epidemiologia y Bioestadistica, Centro de Investigación y de Educación Continua (CENINVEC), Oyameles 30, Colonia La Perla, 57820 Ciudad Nezahualcóyotl, MEX, Mexico

## Abstract

The aims of this study were to evaluate the microbiological quality and the occurrence of nontuberculous mycobacteria (NTM) in a variety of salads and sprouts from supermarkets and street vendors in Mexico City. Aerobic-mesophilic bacteria (AMB) were present in 100% of RTE-salads samples; 59% of samples were outside guidelines range (>5.17 log_10_ CFU per g). Although fecal coliforms (FC) were present in 32% of samples, only 8% of them exceeded the permissible limit (100 MPN/g). Regarding the 100 RTE-sprouts, all samples were also positive for AMB and total coliforms (TC) and 69% for FC. Seven NTM species were recovered from 7 salad samples; they included three *M. fortuitum*, two *M. chelonae*, one *M. mucogenicum*, and one *M. sp.* Twelve RTE-sprouts samples harbored NTM, which were identified as *M. porcinum* (five), *M. abscessus* (two), *M. gordonae* (two), *M. mucogenicum* (two), and *M. avium* complex (one). Most RTE-salads and RTE-sprouts had unsatisfactory microbiological quality and some harbored NTM associated with illness. No correlation between the presence of coliforms and NTM was found. Overall, these results suggest that RTE-salads and RTE-sprouts might function as vehicles for NTM transmission in humans; hence, proper handling and treatment before consumption of such products might be recommendable.

## 1. Introduction

According to the definition given by the FAO and the WHO [1], ready-to-eat (RTE) foods include any comestible that is normally consumed in its raw state. Demand for RTE food has led to an increase in the amount and selection of different products available for the consumers [2]. RTE-salads and RTE-sprouts constitute a suitable and convenient meal for today's lifestyles because they need no cooking or further preparation. As well as being considered low-calorie food, they are rich in fiber and provide a great variety of vitamins, minerals, and other phytochemicals [3]. Their consumption is encouraged in many countries by government health agencies to protect people against a range of illnesses such as cancer and cardiovascular diseases [4]. Therefore, continued increase in the consumption of fresh meals has occurred as a result of efforts to promote better nutrition in the population [4]. As RTE-salads and RTE-sprouts do not need further preparation before consumption, they could potentially contain pathogens that form part of their microflora, posing a public health problem. Fresh vegetables can become contaminated by pathogens as* Salmonella* at any point during the food production process. During preharvest, contact with contaminated irrigation water, soil, manure, or fecal matter of wild animals may occur. These pathogens can both bind to plant leaves and/or be internalized via the leaves or the endophytic root system [5, 6]. During harvest, asymptomatic human carriers might contaminate the products, and at the postharvest level, products become contaminated by contact with polluted water, other asymptomatic human carriers, or the production process environment. Over the last 30 years there has been at least a 24% of increase in the average amount of fresh vegetable consumed per person in the USA [7]. Moreover, the number of gastroenteritis outbreaks caused by foodborne pathogens after consumption of raw vegetables salads and sprouts has increased worldwide [8–11]. Even though* Salmonella* is the most common cause of disease outbreaks associated with lettuce and sprouts [12–15], there are other pathogens (Shiga toxin, producing* E. coli* O157, Norovirus) that have been described as relevant microbial hazards [16–18]. For example, a large outbreak of hemolytic-uremic syndrome caused by STEC O104:H4 linked to sprouts occurred in Germany [19, 20].

Nontuberculous mycobacteria (NTM) are opportunistic pathogens found in the environment that cause life-threatening infections in humans, other mammals, and birds [21, 22]. The incidence of NTM disease is increasing worldwide [23], in both immunocompetent and immunocompromised subjects [22, 24, 25]. As there is no defining evidence for person-to-person transmission for most of the NTM [24, 25], it is therefore important to establish the sources and routes of NTM transmission, since infections can occur through inhalation, ingestion, gastric reflux, or skin trauma [22, 26]. NTM have been isolated from various kinds of food, and many studies support the hypothesis that food, especially raw or partially cooked products, plays a role as a source of NTM for humans, primarily in countries with similar processing food routes and climates [27–29]. The aims of this study were to evaluate the microbiological quality of RTE-salads and RTE-sprouts collected in Mexico City and to analyze the occurrence of NTM in these samples, in order to determine whether these RTE foods may represent a potential risk for NTM infection for the consumers.

## 2. Material and Methods

### 2.1. Area of Study and RTE Food Collection

**Figure 1:**
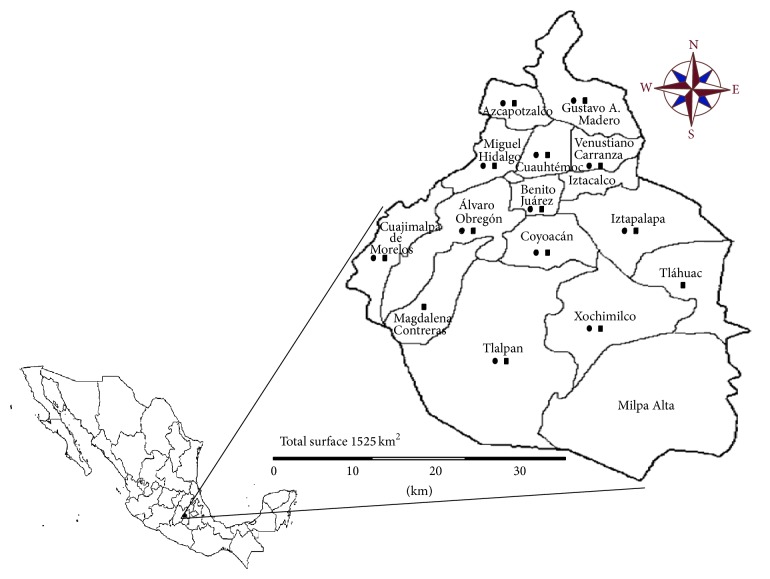
Boroughs of Mexico City where RTE-salads (●) and RTE-sprouts (■) were collected.

The selected area of study was Mexico City, a large urban area that, although it has almost 9 million registered inhabitants, during working hours, reaches a population of nearly 25 million. Mexico City is divided into 16 “delegaciones” or boroughs (Figure 1).  From January to July 2013 a total of 100 RTE-salads samples of raw vegetables (salads from SPM contained mainly lettuce and spinach accompanied by carrot and purple cabbage, while salads from SVS also contained onion, tomatoes, cucumber, Mexican turnip, mushroom, radish, coriander, cactus, and fruits as strawberry, apple, and mango) were collected from different boroughs (Figure 1): 50 samples from different supermarkets (SPM) and 50 from street-vendor stalls (SVS). Also, 100 RTE-sprouts samples (alfalfa, soybean, broccoli, carrot, radish, onion, amaranth, clover, arugula, lentil, wheat, melon, turnip or combinations of alfalfa and broccoli, alfalfa and clover, alfalfa and onion, alfalfa and soybean, and alfalfa and radish) were purchased (Figure 1), from August 2013 to February 2014: 50 from different SPM and 50 from SVS. Salads and sprouts collected from SPM were purchased in bags provided by a bigger food processing company. Those vegetables collected from SVS were prepared directly by vendors at the selling spot. At the moment of purchase the RTE-salads and RTE-sprouts were packaged in sterile plastic containers and were transported to the laboratory for their analysis within 2 h after collection.

### 2.2. Microbiological Analysis

According to the FDA [30], 50 grams from each sample was placed in ethylene oxide gas-sterilized polypropylene bags (Whirl-Pak, Nasco, USA) and 450 mL of lactose broth was added in order to achieve a final dilution 1 : 10 (10^−1^). Samples were homogenized for 2 min in a stomacher (tissue disrupter) and serially diluted (10^−1^–10^−5^); then, these dilutions were used for quantification (CFU/mL) and estimation (MPN/g) of microorganisms. Each sample was tested for the presence of aerobic-mesophilic bacteria (AMB), total coliforms (TC), and fecal coliforms (FC) following the methods approved by the FDA's Bacteriological Analytical Manual [30]. All the data obtained in this work was analyzed according to the official 093 guideline [31], which establishes that food samples should only contain up to 5.17 log_10_ CFU per g (150,000 CFU/g) of AMB and up to 100 MPN/g of FC.

### 2.3. Isolation and Identification of Mycobacteria

For both RTE foods (salads and sprouts), 45 mL of supernatant from dilution 10^−1^ was placed in sterile conical centrifugation tubes (Falcon type) of 50 mL and then was centrifuged (4,000 ×g at 25°C for 20 min). Supernatants were discarded, and pellets were resuspended in 20 mL of 50 ppm chlorine solution or 20 mL of 0.1% cetylpyridinium chloride, for salads and sprouts, respectively. The suspensions were incubated at room temperature for 30 min and then neutralized with 20 mL of phosphate buffer (pH 7.0). Samples were centrifuged as above and pellets were resuspended in 5 mL of Dubos medium (Difco, Becton Dickinson, Sparks, MD) with albumin-dextrose-catalase (ADC; Becton Dickinson, Mexico); 100 *μ*L of this suspension was inoculated onto Middlebrook 7H10 agar (Difco, Becton Dickinson) supplemented with ADC, cycloheximide (500 *μ*g/mL), and the PANTA cocktail (Becton Dickinson) (40 U/mL polymyxin B, 4 *μ*g/mL amphotericin B, 16 *μ*g/mL nalidixic acid, 4 *μ*g/mL trimethoprim, and 4 *μ*g/mL azlocillin). Plates were incubated at 35°C and were examined daily for the first eight days and thereafter once a week for two months. Once the bacterial growth had been observed on the Middlebrook 7H10 agar, the identification of acid-fast bacilli was carried out by Ziehl-Neelsen stain. Acid-fast bacilli were subcultured on Middlebrook 7H10 agar, labeled by sampling location and with a consecutive number.

Strains belonging to the genus* Mycobacterium* and to the* M. tuberculosis* complex were identified by two PCR assays previously described [32]. Briefly, 3 *μ*L aliquots of bacterial lysates were subjected to amplification, using a standard* Taq* polymerase (Life Technologies, Rockville, MD) in a total volume of 50 *μ*L of PCR mixture; RAC1 and RAC8 and MTB-F and MTB-R primers [32] were used for identification of the* Mycobacterium* genus and of the strains belonging to the* M. tuberculosis* complex, respectively. The amplicon produced by the primer combination of RAC1 and RAC8 contains the last 99 codons of the* mur*A gene, the promoter region of the* rrn*A operon, and 360 nucleotides from the 5′ end of the 16S rRNA gene. As shown by Perez-Martinez et al. [33], the amplicon size varies depending on the mycobacteria species, from 934 to 1300 bp. MTB-F/MTB-R primers amplified a DNA fragment coding for the last five codons of* mur*A gene, the promoter region of the* rrn*A operon, and the 5′ end of the 16S rRNA; a 488 bp fragment characteristic only for* M. tuberculosis* complex members should be amplified. Therefore, by exclusion, mycobacteria strains that did not belong to the* M. tuberculosis* complex were considered to be NTM. These NTM species were identified by three methods: (i) PCR restriction enzyme pattern analysis (PRA) of the 65 kDa heat shock protein gene (*hsp*65), as described by Telenti et al. [34]; (ii) sequencing of the hypervariable region 2 (V2) of the 16S rRNA gene [35]; and (iii) sequencing of the* rpo*B gene [36]. Mycobacterial PRA was performed by PCR amplification of a 439 bp fragment of the* hsp*65 gene by using primers Tb11 and Tb12 [34]. PCR products were digested in two separate reactions with two restriction enzymes, BstEII (New England Biolabs) and HaeIII (Invitrogen). Digested products were then analyzed using the Agilent 2100 bioanalyzer. DNA 1000 LabChips (Agilent) were used according to manufacturer's protocol. PRA results were interpreted with the algorithm described by Telenti et al. [34], which is available on the PRA database [37].

Identification of the mycobacterial species was also carried out by automatized sequence of the hypervariable region 2 (V2) of the 16S rRNA gene and of the* rpo*B gene. The amplification of the 16S rRNA gene was performed using the RAC1 and RAC8 primers [32]. For the amplification of the* rpo*B gene, the Myco-F and Myco-R primers were used to obtain a product of 723 bp [36]. Both products of PCR were sequenced using the RAC8 [32] and Myco-F [36] primers, respectively, and the big dye terminator ready reaction kit (Perkin-Elmer, Inc., Wellesley, MA). The sequences were analyzed by ABI PRISM 310 genetic analyzer system (Perkin-Elmer). Nucleotide sequences were compared to known sequences in the GenBank database by using the Blastn algorithm. Species identifications were based on the 100% similarity cut-off for the 16S rRNA gene and ≥97% for the* rpo*B gene.

The identification of some of the isolated NTM was not possible using the methods described above; therefore, full-length 16S rRNA gene was amplified using the 13B/8FPL universal primers [38]. Purified PCR products were directly sequenced in both forward and reverse directions using the same primers as for PCR. Nucleotide sequences were compared to known sequences in the GenBank database by using the Blastn algorithm.

### 2.4. Statistical Analyses

Categorical variables (AMB, TC, and FC) were compared using the (chi)^2^ test for the 2 categories of origin of samples (SPM and SVS). We calculated *P* value from Fisher test for corresponding data. The values of median of CFU of AMB and MPN of TC and FC were compared using the Kruskal-Wallis test. Two-tailed probability values were calculated. The point-biserial correlation coefficient (*r*_pb_) value was calculated to quantify the relationship between the nominal (presence of NTM) and quantitative variables (concentration of AMB, TC, and FC). Similarly, the phi correlation coefficient (*r*_phi_) value was calculated to quantify the relationship between all nominal variables (presence of NTM and AMB, TC, and FC). A *P* value < 0.05 was considered significant. All statistical analysis was run with the Statistical program SPSS for Windows version 21.

## 3. Results

The RTE-salad and RTE-sprout samples had unsatisfactory microbiological quality (results outside acceptable microbiological limits, see reference [39]). A total of 59% of RTE-salads (21% from SPM and 38% from SVS, *P* = 0.001) did not comply with the 093 guideline (see Section 2) (Table 1). In contrast, FC was detected in 32% of samples; of these, only 8% exceeded the permissible limit of the official guideline. The median concentration of AMB in RTE-salad samples from SPM was significantly lower than the one observed in those from SVS (*P* < 0.001). Similarly, we found equivalent results when the median concentrations of MPN/g of TC and FC were compared (*P* < 0.001).

AMB and TC were also present in 100% of RTE-sprouts samples analyzed (Table 1); FC were present in 69% of samples. The median concentration of FC was significantly lower in RTE-sprouts from SPM (3.3 MPN/g) than the one found in SVS (23.5 MPN/g) (*P* = 0.007). However, the median concentrations of AMB in samples from SPM and SVS were similar (*P* = 0.762). Likewise, we observed the same result when we compared the median concentration values of TC between samples from SPM and from SVS (*P* = 0.169).

One hundred RTE-salads were tested for the presence of mycobacteria. Seven RTE-salads harbored NTM: three were* M. fortuitum*, two were* M. chelonae*, one was* M. mucogenicum,* and one was* M. sp *(Table 2). All NTM were isolated from RTE-salads collected in SVS (*P* < 0.05). No correlation between the presence of NTM and the presence of AMB (*r*_phi_ = 0.154, *P* = 0.234), TC (*r*_phi_ = 0.056, *P* = 1.000), and FC (*r*_phi_ = 0.064,  *P* = 0.453) was found. Likewise, no correlation between the presence of NTM and the number of AMB (*r*_pb_ = 0.098,   *P* = 0.330), TC (*r*_pb_ = −0.024, *P* = 0.812), and FC (*r*_pb_ = 0.162, *P* = 0.106) was observed. Of the 100 RTE-sprout samples analyzed, 12 yielded NTM.* M. porcinum* was the most frequently isolated organism (five isolates). We have also recovered two strains of* M. abscessus*, two of* M. gordonae*, two of* M. mucogenicum*, and one strain belonging to the* M. avium* complex (Table 2). Comparison of the number and species of NTM isolated from SPM and SVS showed no significant differences (*P* = 0.424). We also found no correlation between the presence of NTM and the presence of AMB, TC, and FC (*r*_phi_ = −0.081; *P* = 0.475) in RTE-sprouts. Similarly, we did not observe correlation between the presence of NTM and the median concentration of TC (*r*_pb_ = 0.046, *P* = 0.653) and FC (*r*_pb_ = 0.099, *P* = 0.326). Nevertheless, we did observe a significant correlation between the presence of NTM and the median concentration of AMB (*r*_pb_ = 0.331; *P* = 0.001).

## 4. Discussion

This study shows that AMB were detected in all RTE-salads regardless of source, with limits ranging from 3 to 6.6 log_10_ CFU/g. AMB counts were found to be higher than those reported for RTE-salads in Johannesburg, South Africa [40]. In contrast, the number of these microorganisms (AMB) was found to be lower than that reported for RTE-salads from Porto, Portugal, and Catalonia, Spain [41, 42]. The frequency of TC on RTE-salads observed here coincides with previous studies [43–45] carried out both in Mexico and in Brazil, countries where proper raw vegetable product handling and sanitation practices need to be promoted and implemented.

FC were identified in 32% of RTE-salad samples; their frequency was substantially lower than that reported for RTE-salads from other developing countries. For instance, frequencies of 90.5% and 89% of FC were reported from Brazil [45] and Costa Rica [46], respectively. In spite of that, Gómez-Aldapa et al. [43] and Castro-Rosas et al. [44] reported frequencies of FC of 95.5% and 99%, respectively, collected from restaurants in our country. We can assume that these higher numbers are the result of the greater number of people that can possibly be involved in the handling of this type of food in these types of places. Furthermore, these different numbers compared to the ones found by our work may be due also to the quality of water used for growing those vegetables, to the different methods of handling them, and to the different sampling techniques used. Unsatisfactory microbiological state of RTE-salads was more frequently observed in salads from SVS than in those from SPM (*P* = 0.001); we suggest that these SVS lack adequate appreciation of basic food safety issues; that is, street vendors keep the salads at room temperature (up to 27°C) unlike supermarkets, where the salads are stored at refrigerated temperatures (4 to 7°C). Street vendors often use stands that are of inefficient construction; running water is not easily accessible and hand and dish washing are performed in the same bucket, sometimes without soap. Wastewater is usually discarded right there in the streets, and garbage is likewise “conveniently” discarded right next to the stands, providing attraction, food, and harborage for insects and rodents. In many cases, toilets are not available, thus forcing the vendors to eliminate their body wastes also in areas close by and to return to their vending sites without washing their hands. Such conditions and practices are likely to lead to cross contamination of street food. In other cases, vendors buy raw materials from dubious sources, and these materials may be contaminated with foodborne pathogens [47–49].

Regarding the microbiological quality of the sprouts collected in our work, the frequencies and concentrations of AMB found coincide with previous studies from different countries [3, 42, 50]. It has been reported that vegetable seeds could contain <2 log_10_ CFU/g of BMA. This naturally occurring population of microorganisms can rapidly increase during germination and sprouting because of the favorable conditions for bacterial growth [3]. Consequently, if seeds become contaminated with a pathogen, the sprouting process provides excellent conditions for consequent growth and distribution.

In our study, TC were detected in 100% of sprouts samples, result which coincides with that reported for bean sprouts in Central Mexico, where* Salmonella* and diarrheagenic* Escherichia coli* pathotypes were also identified [51]. On the other hand, FC were detected in the 69% of our RTE-sprout samples; this frequency was found to be lower than that reported for RTE-sprouts from markets in the town of Pachuca, Mexico [51].

Those high frequencies and concentrations of AMB, TC, and FC found in our RTE-sprouts may mean that they are so heavily contaminated that traditional sanitation practices would not be enough to reduce bacterial contamination. It has been suggested that an alternative, which can be used only by big food companies, for reducing the bacterial load in sprouts is the use of ionizing radiation, since it has been reported that a dose of 1.5 and 2 kGy can significantly reduce* E. coli* O157:H7 and* Salmonella* to nondetectable limits in bean and radish sprouts [52]. Therefore, our results conclude, together with some others [43, 44, 51] reported for our country, the highlighted need for implementing stricter hygienic control standards and measures for vegetables and sprouts grown in Mexico.

In this study, we combined conventional and molecular methods and detected and identified NTM in 7 RTE-salads and 12 RTE-sprouts. All NTM identified in this study have also been found in Mexican water samples [53] and in water samples from other countries [21, 54]. Therefore, it is possible that water may be the original source of NTM transmission to vegetables (used in the salads) or sprouts while growing or during harvesting, washing, slicing, soaking, packaging, and preparation. NTM are opportunistic pathogens found in water and soil as normal flora. Therefore, another possibility of the presence of NTM in RTE-salads or RTE-sprouts comes from the soil and the water used for the growing and the irrigation, respectively, of those vegetables.

A significant correlation between the presence of NTM and the median concentration of AMB (*P* = 0.001) was observed. This may be due to the fact that NTM as well as AMB are environmental organisms that have features in common, such as, growing in moderate to warm temperatures (20 and 45°C) and in aerobiosis conditions.

Some of the NTM identified in our study included species that have been frequently associated with human illness in other countries, that is,* M. avium*,* M. fortuitum*,* M. abscessus*,* M. chelonae*, and* M. mucogenium *[55, 56]. In the case of* M. avium, *it has been suggested that the most common portals of entry of this microorganism are the gastrointestinal and the respiratory tracts [57, 58]. In AIDS patients,* M. avium* is acquired predominantly via the gastrointestinal tract, where it is able to invade the intestinal mucosa, to infect and multiply within submucosal macrophages, and to cause bacteremia, leading to the dissemination of the microorganism to the liver, spleen, and bone marrow [58]. Regarding* M. mucogenium,* it has been associated with infection of the gastrointestinal tract in patients with a diagnosis of Crohn's disease [59].

In Mexico City, the prevalence of NTM infections is poorly known and only a few studies have been published. Among these studies, Lopez-Alvarez et al. [60] reported in 2010 that 15% of mycobacterial strains isolated from 67 HIV patients belonged to NTM (10 strains were identified as* M. avium* and 1 strain was identified as* M. intracellulare*). In another recent study, Cortés-Torres et al. in 2013 [61] reported that 37% of 96 patients in a Mexico City Hospital, suffering from various immunodeficiencies, presented several strains of NTM, including* M. avium*,* M. simiae*,* M. gordonae,* and* M. kansasii*. Although no combined conclusion could be reached between these findings and ours, further studies of DNA fingerprinting of NTM should be carried out, in order to confirm that NTM isolated from RTE-salads and RTE-sprouts are the same as those isolated from patients.

## 5. Conclusions

Most RTE-salads and RTE-sprouts analyzed in this study had unsatisfactory microbiological quality and some harbored NTM associated with illness. Measures to diminish or eliminate NTM strains from these food items might be advisable, such as a proper handling and washing before consumption of these products. RTE-salads and sprouts could be considered as potential sources for NTM infections in humans.

## Figures and Tables

**Table 1:** Populations and frequencies of aerobic-mesophilic bacteria (AMB), total coliforms (TC), and fecal coliforms (FC) on RTE-salads and RTE-sprouts samples.

Microorganisms group	Minimum	Median	Maximum	Frequency (%)	Number of samples out of the 093 guideline^*^ (%)
RTE-salads from SPM^a^					
AMB	3	4.9	6.6	50 (100)	21 (42)
TC	<3	56	>1100	46 (92)	NA
FC	<3	<3	210	7 (14)	2 (4)
RTE-salads from SVS^a^					
AMB	3	6.1	6.7	50 (100)	38 (76)
TC	3	1100	>1100	50 (100)	NA
FC	<3	1.5	>1100	25 (50)	6 (12)
RTE-sprouts from SPM^a^					
AMB	6.1	7.4	8	50 (100)	NA
TC	6.1	460	>1100	50 (100)	NA
FC	<3	3.3	>1100	29 (58)	NA
RTE-sprouts from SVS^a^					
AMB	6.1	7.3	8.8	50 (100)	NA
TC	26	1100	>1100	50 (100)	NA
FC	<3	23.5	>1100	40 (80)	NA

^a^ *n* = 50. Minimum, median, and maximum values are in log_10_⁡ CFU per g for aerobic-mesophilic bacteria and in most probable number (MPN) per g for total coliforms and fecal coliforms. SPM: supermarkets, SVS: street-vendor stalls, and NA: not applicable (there is no official guideline for this food).

^*^Guideline that establishes that food samples should contain up to 5.17 log_10_⁡ CFU per g (150,000 CFU/g) of AMB and up to 100 MPN/g of FC.

**Table 2:** Characteristics of positive samples for NTM and species identified.

Foods	Origin	Number of positive samples, type	Ingredients	Number and species of NTM identified
RTE-salads	SVS	3, mixed	Lettuce, carrot, cucumber, Mexican turnip, tomatoes, onion	2, *M. fortuitum* 1, *M. chelonae *
	SVS	1, mango	Lettuce, mango, Mexican turnip, strawberry	1, *M. fortuitum *
	SVS	3, nopal	Cactus, tomatoes onion, coriander	1, *M. mucogenicum* 1, *M. chelonae* 1, *M. sp *

RTE-sprouts	SPM	3, alfalfa	Alfalfa sprouts	2, *M. abscessus* 1, *M. gordonae *
	SPM	1, alfalfa and clover	Alfalfa sprouts Clover sprouts	1, *M. porcinum *
	SPM	1, alfalfa and onion	Alfalfa sprouts onion sprouts	1, *M. porcinum *
	SVS	4, alfalfa	Alfalfa sprouts	1, *M. avium *complex 3, *M. porcinum *
	SVS	1, alfalfa and soybean	Alfalfa sprouts soybean sprouts	1, *M. mucogenicum *
	SVS	1, soybean	Soybean sprouts	1, *M. mucogenicum *
	SVS	1, broccoli	Broccoli sprouts	1, *M. gordonae *

SVS: street-vendor stalls; SPM: supermarket.
